# 3D-TCM-Driven Bit-Level Image Encryption via S-Box Feedback Algorithm

**DOI:** 10.3390/e28050535

**Published:** 2026-05-08

**Authors:** Jie Zhang, Wenjie Zhou, Mingxu Wang, Yiting Lin

**Affiliations:** 1 School of Electronics and Information Engineering, Lanzhou Jiaotong University, Lanzhou 730070, China; wjzhou@lzjtu.edu.cn; 2 College of Information Engineering, Dalian Ocean University, Dalian 116023, China; 15041119328@163.com; 3 University of Electronic Science and Technology of China Zhongshan Institute, Zhongshan 528406, China; yitinglin@ieee.org; 4 University of Electronic Science and Technology of China, Chengdu 611731, China

**Keywords:** chaos-based encryption, hyperchaotic system, S-box construction, secure image encryption

## Abstract

Most existing low-dimensional chaotic maps suffer from a limited dynamical complexity and dynamic degradation, which restrict their effectiveness in image encryption. To address this issue, a novel three-dimensional chaotic map (3D-TCM) was constructed to improve dynamical complexity and stability, and its superiority was verified through a dynamical analysis. Based on these advantages, a plaintext-related image encryption scheme was designed by combining bit-level permutation and S-box-based diffusion. The experimental results show that the proposed scheme achieved high information entropy, a low pixel correlation, and desirable NPCR and UACI values, and successfully passed NIST SP800-22 statistical tests, demonstrating a strong resistance to differential attacks and overall robustness.

## 1. Introduction

In our era of information explosion, digital images have become a primary medium for communication and data exchange. However, transmitting image data over open and insecure networks exposes them to unauthorized access and potential attacks, making robust image encryption techniques essential [1]. With the rapid growth of multimedia applications and networked environments, the demand for efficient and secure image protection mechanisms has become increasingly critical.

Chaos-based image encryption algorithms (IEAs) may provide an effective solution due to the inherent properties of chaotic maps (CMs), such as sensitivity to initial conditions, pseudo-randomness, and nonlinear dynamics, which naturally satisfy Shannon’s requirements of confusion and diffusion [2,3]. Low-dimensional chaotic maps (LDCMs), such as the Sinesine map, are widely adopted in IEAs due to their simple structure and low computational cost. Based on LDCMs, numerous IEAs have been proposed [4,5,6,7]. To further improve the performance, various enhanced schemes based on LDCMs and their variants have also been investigated, including optimization-based methods, hybrid chaotic structures, and permutation–diffusion frameworks [8,9,10,11,12,13,14]. However, recent studies have indicated that many LDCMs suffer from a limited dynamical complexity, a small key space, and dynamic degradation under finite precision, which may weaken their resistance to cryptanalysis. Such limitations restrict their ability to generate sufficiently complex chaotic sequences, thereby affecting the overall security performance of the corresponding encryption schemes. In contrast, high-dimensional chaotic maps (HDCMs) exhibit richer dynamical behaviors, broader parameter spaces, and higher unpredictability, making them more suitable for secure image encryption. Accordingly, recent research has increasingly focused on constructing high-dimensional or hybrid CMs to enhance the security performance.

Numerous HDCM-based IEAs have been developed. Lai et al. proposed a fast parallel multi-image encryption scheme based on a dual-memristor CM, achieving a high efficiency and strong resistance to statistical and differential attacks [15]. Kareem et al. introduced an algebraically enhanced three-dimensional (3D) CM combined with hash-based initialization, significantly improving the nonlinear dynamics and overall encryption robustness [16]. Aibai et al. developed an enhanced AES framework driven by a 2D HDCM, demonstrating that integrating chaos with classical cryptography can effectively strengthen security mechanisms [17]. Duan et al. designed a multi-image encryption scheme based on an HDCM and dual S-boxes, enhancing both the encryption capacity and the resistance to various attacks [18]. Deepankumar et al. employed a 3D CM for bit-plane-level permutation and diffusion, showing an improved key sensitivity, a reduced pixel correlation, and a strong robustness against multiple attack scenarios [19]. Kocak et al. proposed a 2D HDCM with superior dynamical properties, which significantly enhances the confusion and diffusion capabilities of the IEA [20]. Liu et al. further introduced a 2D non-degenerate CM-based method to batch-generate high-nonlinearity keyed S-boxes while eliminating fixed points and short cycles, thereby enhancing the resistance to cryptographic attacks [21]. Despite these advances, many existing HDCM-based IEAs still suffer from several limitations, such as an insufficient key sensitivity, limited resistance to differential attacks, and reliance on fixed or deterministic traversal structures. In addition, the interaction between chaotic dynamics and encryption operations is often not sufficiently tight, which limits the effective utilization of chaotic properties in practical encryption processes. Moreover, the use of static or linearly driven chaotic sequences derived from CMs without adaptive control may reduce randomness and degrade the security performance in practical implementations.

Therefore, the key challenge lies in jointly improving the dynamical quality of CMs and their effective integration into IEAs. Specifically, it remains difficult to design CMs with a sufficiently high complexity and stability while simultaneously constructing encryption frameworks that can fully exploit these properties to achieve a strong key sensitivity and diffusion capability. Motivated by this, this paper proposes a three-dimensional trigonometric coupled map (3D-TCM) with enhanced chaotic properties and stability and further constructs a plaintext-related IEA (TCM-IEA) based on it. By tightly coupling chaotic sequence generation with encryption operations, the proposed IEA enhances both the randomness and security performance. The main contributions are summarized as follows:

1. A 3D-TCM was constructed that exhibits a wide chaotic range, multiple positive Lyapunov exponents, and a strong dynamical complexity, providing a reliable foundation for secure image encryption.
2. A plaintext-related TCM-IEA was proposed by integrating bit-level conditional permutation and S-box driven feedback diffusion, where the key generation mechanism combines SHA-512 and CMs to enhance the randomness and key sensitivity.
3. Extensive experiments and security analyses indicated that the TCM-IEA exhibits a high performance in statistical properties, differential resistance, and robustness against noise and data loss attacks, confirming its effectiveness and practical applicability.

The remainder of the study is organized as follows: Section 2 constructs the 3D-TCM and analyzes its chaotic properties. Section 3 details the TCM-IEA framework, covering key generation, bit-level conditional permutation and S-box driven feedback diffusion and following a decryption process. Section 4 evaluates the security and performance of the TCM-IEA through several classic attacks. Section 5 provides a concluding summary.

## 2. Related Work

### 2.1. Construction of the 3D-TCM

The sine map is a classical one-dimensional CM, defined by [22]:

(1) $$x_{n+1}=\mu \sin(\pi x_{n})/4,$$

where μ is the control parameter. $x_{n}$ and $x_{n+1}$ are the state variables of the CM at iteration n and n+1.

Although the sine map has a simple structure, its chaotic behavior exists only within a limited parameter range, which restricts its applicability in IEAs. To improve this issue, various coupling strategies have been investigated for CMs. For example, Gao et al. extended the sine–logistic CM by incorporating an exponential function [23]. Wang et al. constructed a 3D CM through a combination of sine and linear functions [24]. Wang et al. further enhanced the 3D sine-based CM by incorporating fractional-order calculus, thereby achieving a wider chaotic range [25]. However, these CMs rely heavily on linear combinations or basic algebraic operations, which limit their nonlinear expressive capability.

To expand the chaotic range, the 3D-TCM is defined as follows:

(2) $$\left\{\begin{array}{c} x_{n+1}=(a\cdot \sin(\pi x_{n})+b\cdot \cos(\pi y_{n}))\mathrm{mod}\;1\\ y_{n+1}=(a\cdot \sin(\pi y_{n})+b\cdot \cos(\pi z_{n}))\mathrm{mod}\;1\\ z_{n+1}=(a\cdot \sin(\pi z_{n})+b\cdot \cos(\pi x_{n}))\mathrm{mod}\;1 \end{array}\right.,$$

where a and b are control parameters. $x_{n}$, $y_{n}$, and $z_{n}\in [0,1)$ are the state variables of the 3D-TCM at iteration n, while $x_{n+1}$, $y_{n+1}$, and $z_{n+1}$ represent the states at the next iteration. The symbol n denotes the iteration index. The mod denotes the modulo operation that maps the state variables into the interval [0,1). To verify the chaotic properties of the 3D-TCM, we analyzed its dynamical behavior through a bifurcation diagram, phase trajectories, Lyapunov exponents, sample entropy, NIST tests, and a 0–1 test.

### 2.2. Attractor Analysis of the 3D-TCM

To further investigate the dynamical behavior of the 3D-TCM, this section analyzes its attractor behavior under different control parameters. By varying a and b and examining the evolution trajectories of the state variables $x_{n}$, $y_{n}$, and $z_{n}$, the geometric structures of the 3D-TCM in the 3D phase space can be clearly revealed.

**Figure 1:**
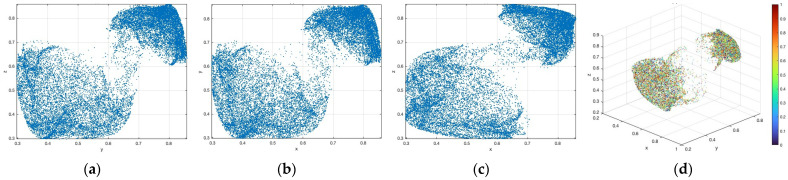
Attractor of the 3D-TCM when (a,b)=(0.8,0.1). (**a**) y-z plane (**b**) x-y plane (**c**) x-z plane (**d**) x-y-z plane.

**Figure 2:**
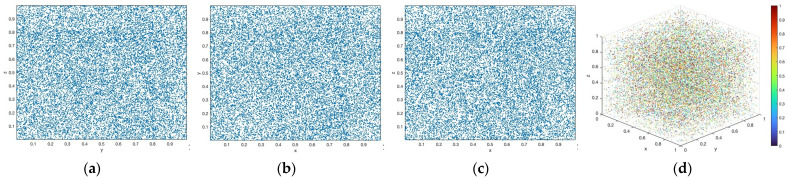
Attractor of the 3D-TCM when (a,b)=(3,3). (**a**) y-z plane (**b**) x-y plane (**c**) x-z plane (**d**) x-y-z plane.

Figure 1 and Figure 2 present the attractors corresponding to these parameter sets. For each case, the projections on the Y-Z, X-Y, X-Z, and X-Y-Z planes, as well as the 3D phase trajectories, are illustrated. From Figure 1, when (a,b)=(0.8,0.1), the 3D-TCM exhibits a typical structured chaotic attractor with a non-uniform density distribution, indicating strong nonlinear coupling among the state variables. As shown in Figure 2, when (a,b)=(3,3), the trajectories are distributed almost uniformly in the state space, suggesting that the 3D-TCM approaches a higher level of chaotic complexity and mixing behavior. The results indicate that the 3D-TCM can obtain a wide variety of chaotic attractors with distinct geometric structures. The 3D-TCM exhibits a strong sensitivity to parameter variations and initial conditions, as well as good phase space coverage and complexity.

### 2.3. Bifurcation Diagram

**Figure 3:**
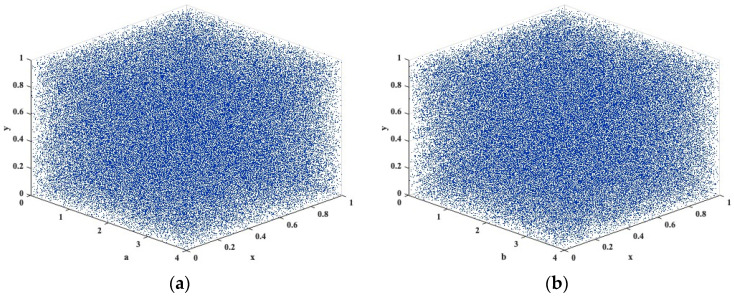
BD of the 3D-TCM: (**a**) *a* and (**b**) *b*.

A bifurcation diagram (BD) visualizes the dynamical behavior of a CM as the control parameter varies, revealing transitions from periodic motion to chaotic states [26]. As demonstrated in Figure 3, the 3D-TCM exhibits complex dynamical evolution over a wide parameter range. It is important to acknowledge that the BD reflects the CM’s dynamical evolution rather than the statistical uniformity of trajectory distributions. Consequently, the nonuniform distribution of points in the BD is a common phenomenon in CMs and does not affect their chaotic properties.

### 2.4. Lyapunov Exponent

The Lyapunov exponent (LE) quantifies the average exponential divergence rate of nearby trajectories in phase space and is widely used to characterize dynamical instability. In general, a positive LE indicates sensitive dependence on initial conditions, while the presence of multiple positive LEs suggests higher-dimensional complex dynamics, often associated with hyperchaotic characteristics. Furthermore, larger LE values generally correspond to stronger divergence behavior. The LEs can be calculated via [27]:

(3) $$\lambda =\underset{n\to \infty }{\lim}\frac{1}{n}\sum_{i=0}^{n-1}\ln\frac{d_{i}}{\delta_{0}},$$

where λ refers to the largest LE. n specifies the total number of iterations, i denotes the iteration index, $d_{i}$ represents the Euclidean distance between two nearby trajectories at iteration i, and $\delta_{0}$ is the initial perturbation magnitude.

**Figure 4:**
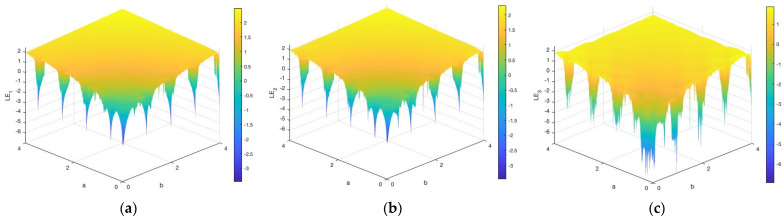
LE results of the 3D-TCM: (**a**) $LE_{1}$, (**b**) $LE_{2}$, and (**c**) $LE_{3}$.

As demonstrated in Figure 4, the 3D-TCM exhibits multiple positive LEs over a wide parameter range, indicating that the system possesses strong chaotic behavior and tends toward hyperchaotic dynamics in certain regions. In particular, the distributions of the three LEs over the (a,b) parameter plane reveal that the positive regions are not uniformly distributed, but appear in multiple disjoint intervals. This indicates that the 3D-TCM exhibits multi-regime dynamics with a strong sensitivity to initial conditions and complex divergence across multiple parameter sub-regions, enhancing the randomness and unpredictability in practical applications.

### 2.5. Sample Entropy

**Figure 5:**
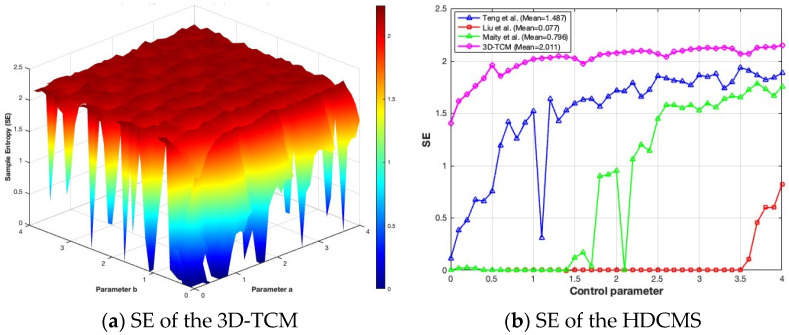
The SE of the 3D-TCM and the comparison with HDCMs [26,29,30].

As posited by [28], sample entropy (SE) is a measure of the degree of disorder or unpredictability in a time series. A larger SE value indicates a higher randomness and a closer proximity to true randomness, which is a desirable property for key stream generation. It is imperative to note that, as the SE value of a CM increases, the difficulty of prediction also increases. In this section, the SE is computed by:

(4) $$SE(m,r,N)=-\log\frac{S}{P},$$

where m is the template dimension. r and N are the similarity threshold and the length of the time series, respectively. As demonstrated in Figure 5, the 3D-TCM exhibits consistently high SE values over a wide range of control parameters (a,b), with relatively small variation. The average SE value is significantly high, indicating that the 3D-TCM can generate sequences with a strong dynamical complexity. Furthermore, the regions with higher SE values are consistent with those where positive LEs are observed, suggesting that the 3D-TCM possesses an excellent randomness generation capability. As further observed from Figure 5b, compared with conventional HDCMs [26,29,30], the 3D-TCM maintains higher SE values over a broader parameter range with smoother variation, indicating more stable chaotic dynamics and a stronger capability in generating pseudo-random sequences with an enhanced unpredictability and complexity.

### 2.6. NIST Test

The NIST SP800-22 comprises 15 statistical tests that are employed to detect non-randomness in binary sequences [31]. As demonstrated in Table 1, the results of all the tests indicate p-values that exceed the 0.01 significance threshold. This indicates that there is no rejection of the null hypothesis of randomness across all dimensions. It indicates that the sequences generated by the 3D-TCM exhibit a high degree of statistical randomness.

### 2.7. Gottwald-Melbourne 0–1 Test

**Figure 6:**
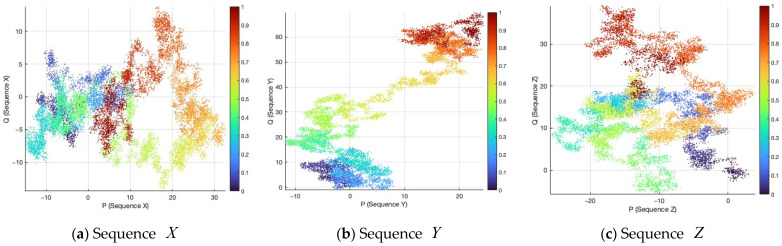
Gottwald–Melbourne 0–1 test results when (a,b)=(3.2,2.7).

To assess the chaotic sequences produced by the 3D-TCM, the 0–1 chaos test introduced by Gottwald and Melbourne was employed. In this method, the time series is mapped to a two-dimensional random walk. When the trajectory shows Brownian motion-like behavior, the sequence is considered random. The corresponding results are shown in Figure 6. It can be seen that the trajectories exhibit typical Brownian motion patterns, suggesting that the generated sequences have strong chaotic properties and a good randomness.

## 3. Encryption and Decryption Procedures

**Figure 7:**
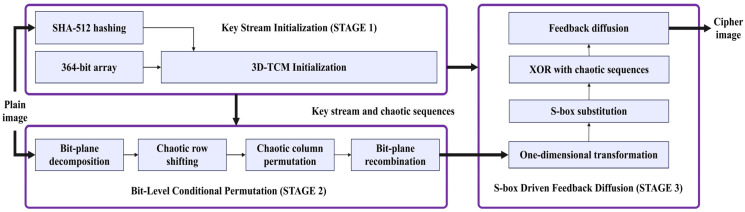
Block diagram of the TCM-IEA.

The employment of the 3D-TCM leverages its high sensitivity to initial conditions and control parameters, thereby ensuring the initialization of highly unpredictable pseudo-random sequences. This improves the resistance against attacks. The overall architecture of the TCM-IEA is depicted in Figure 7. The framework consists of three main modules: key generation, bit-level conditional permutation, and S-box-driven feedback diffusion. In the key stage, the plaintext image and the 3D-TCM are jointly utilized to generate the plaintext-related parameters and chaotic sequences required for encryption. Then, these sequences are used to control the permutation and diffusion processes. The remaining part of this section describes the specific TCM-IEA processes.

### 3.1. Generation of Keystream

For the plaintext image PI, a hash-based key generation mechanism is first applied to ensure plaintext sensitivity. The SHA-512 hash function is utilized to transform the image into a 128-character hexadecimal string $K_{\mathrm{PI}}$, where each hexadecimal digit represents 4 bits. Then, 18-decimal values are calculated as follows:

(5) $$HV_{\mathrm{PI}}=hex2dec(K_{\mathrm{PI}}),$$

where hex2dec(⋅) converts $K_{\mathrm{PI}}$ into the corresponding decimal values, which then undergo a modulus operation so that the resulting values lie within [0,255]. Thereafter, two parameters FG and SG are calculated based on $HV_{\mathrm{PI}}$:

(6) $$\left\{\begin{array}{c} FG=round(mean(HV_{\mathrm{PI}}(1:9)))\\ SG=round(mean(HV_{\mathrm{PI}}(10:18))) \end{array}\right.,$$

where mean(⋅) represents the average value. round(⋅) denotes the rounding operation to the nearest integer. These values are then used to adaptively update the initial states and control parameters of the 3D-TCM. Given the preset parameters (a, b, $x_{0}$, $y_{0}$, $z_{0}$), the final parameters are obtained through a 364-bit random binary string $K_{B}$ constructed following the IEEE 754 floating-point format, and this is then combined with the initial values and the control parameters using Algorithm 1 [32]:

(7) $$x=\frac{\sum_{i=1}^{52}b_{i}2^{52-i}}{2^{52}},$$

where $b_{i}\in (0,1)$ corresponds to the i-th bit of the binary key sequence $K_{B}$, and i represents the corresponding bit index. The 52-bit precision reflects the mantissa length of the IEEE 754 double-precision floating-point format, enabling the binary sequence to be converted into a real value within the interval (0,1).
**Algorithm 1.** Computational workflow of secret keys**Input**:
$K_{B}$,
a,
b$,\;x_{0}$$,\;y_{0}$$,\;z_{0}$, FG, SG**Output**:
$a^{\prime }$,
$b^{\prime }$$,\;x_{0}^{\prime }$$,\;y_{0}^{\prime }$$,\;z_{0}^{\prime }$$,\;offset_{M}$$,\;offset_{N}$$1:\;a^{\prime }=a+(\sum_{i=1}^{52}K_{B}[i]\times 2^{i-1}/2^{52})\mathrm{mod}(4-a)$$2:\;b^{\prime }=b+(\sum_{i=53}^{104}K_{B}[i]\times 2^{i-53}/2^{52})\mathrm{mod}(4-b)$$3:\;x_{0}^{\prime }=x_{0}+(\sum_{i=105}^{156}K_{B}[i]\times 2^{i-105}/2^{52})\mathrm{mod}(1-x_{0})$$4:\;y_{0}^{\prime }=y_{0}+(\sum_{i=157}^{208}K_{B}[i]\times 2^{i-157}/2^{52})\mathrm{mod}(1-y_{0})$$5:\;z_{0}^{\prime }=z_{0}+(\sum_{i=209}^{260}K_{B}[i]\times 2^{i-209}/2^{52})\mathrm{mod}(1-z_{0})$$6:\;offset_{M}=round(FG+(\sum_{i=261}^{312}K_{B}[i]\times 2^{i-261}/2^{52})\mathrm{mod}256)$$7:\;offset_{N}=round(SG+(\sum_{i=313}^{364}K_{B}[i]\times 2^{i-313}/2^{52})\mathrm{mod}256)$

Based on the updated parameters $a^{\prime }$, $b^{\prime }$, $x_{0}^{\prime }$, $y_{0}^{\prime }$, and $z_{0}^{\prime }$, three chaotic sequences X, Y, and Z and their corresponding integer forms $X_{\mathrm{int}}$, $Y_{\mathrm{int}}$, and $Z_{\mathrm{int}}$ are generated. The integer sequences are obtained through quantization:

(8) $$\left\{\begin{array}{c} X_{\mathrm{int}}=floor(X\times 10^{15})\mathrm{mod}256\\ Y_{\mathrm{int}}=floor(Y\times 10^{15})\mathrm{mod}256\;\;\\ Z_{\mathrm{int}}=floor(Z\times 10^{15})\mathrm{mod}256 \end{array}\right.,$$

where floor(⋅) denotes the floor operation that maps a real value to the largest integer not greater than it.

From the generated sequences, three key streams used in the diffusion stage are extracted according to the hash-dependent offsets $HV_{\mathrm{PI}}$:

(9) $$\left\{\begin{array}{c} CM_{{\mathrm{diff}}_{1}}=X_{\mathrm{int}}(1+HV_{\mathrm{PI}}(2):M\times N+HV_{\mathrm{PI}}(2))\\ CM_{{\mathrm{diff}}_{2}}=Y_{\mathrm{int}}(1+HV_{\mathrm{PI}}(3):M\times N+HV_{\mathrm{PI}}(3))\;\\ CM_{{\mathrm{diff}}_{3}}=Z_{\mathrm{int}}(1+HV_{\mathrm{PI}}(4):M\times N+HV_{\mathrm{PI}}(4)) \end{array}\right.,$$

where M and N denote the dimensions of the plaintext image PI.

This design ensures that the generated key stream is highly sensitive to the plaintext and exhibits a strong randomness.

### 3.2. TCM-IEA Process

The detailed procedure of TCM-IEA employs a permutation–diffusion framework that modifies both the positions and values of the pixels.

#### 3.2.1. Bit-Level Conditional Permutation

Bit-level permutation starts with the original image PI. The process commences with the decomposition of PI into 8 bit-planes $BP=\left\{b_{1},b_{2},\ldots ,b_{8}\right\}$. This is followed by organizing them into two bit-matrices, where the chaotic row-shifting operation and the chaotic column-reordering operation are performed using sequence X, Y, and Z along with the hash key $HV_{\mathrm{PI}}$. The results of these operations are reshaped back into 8 bit-planes using chaotic indices and reordered before being fused together into the final scrambled image $PI_{\mathrm{CON}}$. All permutation operations are performed on discrete bit arrays extracted from pixel values. In this context, the circular shift operator cirshift(⋅) denotes an index-based rotation of finite-length vectors rather than an operation on continuous chaotic signals. The detailed operation is presented in Algorithm 2.
**Algorithm 2.** Bit-level conditional permutation**Input**:
BP$,\;X_{\mathrm{INT}}$$,\;Y_{\mathrm{INT}}$$,\;HV_{\mathrm{PI}}$**Output**:
$PI_{\mathrm{CON}}$$1:\;CM_{1}\leftarrow reshape(X_{\mathrm{INT}}[HV_{\mathrm{PI}}(1)+1:HV_{\mathrm{PI}}(1)+MN],M,N)\mathrm{mod}256$$2:\;CM_{\mathrm{BP}(i)}\leftarrow bitget(CM_{1},i),\mathrm{where}\;i=1,2,\ldots ,8$$3:\;CM_{2}\leftarrow reshape(X_{\mathrm{INT}}[HV_{\mathrm{PI}}(7)+1+MN:HV_{\mathrm{PI}}(7)+5MN],2M,2N)$$4:\;CM_{3}\leftarrow reshape(Y_{\mathrm{INT}}[HV_{\mathrm{PI}}(8)+1:HV_{\mathrm{PI}}(8)+4MN],2M,2N)$$5:\;idx_{1}\leftarrow \arg_{\mathrm{sort}}(X_{\mathrm{INT}}[HV_{\mathrm{PI}}(1)+1:HV_{\mathrm{PI}}(1)+8])$$6:\;G_{1}\leftarrow reshape([BP(idx_{1}(1:4))],2,2)$$;\;G_{2}\leftarrow reshape([BP(idx_{1}(5:8))],2,2)$$7:\;H_{1}\leftarrow reshape([CM_{\mathrm{BP}}(idx_{1}(1:4))],2,2)$$;\;H_{2}\leftarrow reshape([CM_{\mathrm{BP}}(idx_{1}(5:8))],2,2)$$8:\;II\leftarrow X_{\mathrm{INT}}[5MN+1:5MN+2M]$$;\;LL\leftarrow X_{\mathrm{INT}}[5MN+2M+1:5MN+4M]$9: for i=1 to 2M do$10:\;\;\;\;\;s_{1}\leftarrow II\;\mathrm{mod}\;2N$$;\;s_{2}\leftarrow LL\;\mathrm{mod}\;2N$$11:\;\;\;\;\;dir_{1}\leftarrow (-1)(\sum H_{1}(i,:)\mathrm{mod}2)\times (s_{1}+HV_{\mathrm{PI}}(5))$$12:\;\;\;\;\;dir_{2}\leftarrow (-1)(\sum H_{2}(i,:)\mathrm{mod}2)\times (s_{2}+HV_{\mathrm{PI}}(6))$$13:\;\;\;\;\;K_{1}\leftarrow circshift(G_{1}(i,:),dir_{1})$$;\;K_{2}\leftarrow circshift(G_{2}(i,:),dir_{2})$14: **end for**15:for j=1 to 2N do$16:\;\;\;\;\;ord_{CM_{2}}\leftarrow \arg_{\mathrm{sort}}(CM_{2}(:,j),‘\mathrm{descend}’)\;\mathrm{if}\sum H_{1}(:,j)\;\mathrm{odd}\;\;‘\mathrm{ascend}’$$17:\;\;\;\;\;ord_{CM_{3}}\leftarrow \arg_{\mathrm{sort}}(CM_{3}(:,j),‘\mathrm{descend}’)\;\mathrm{if}\sum H_{2}(:,j)\;\mathrm{odd}\;\;‘\mathrm{ascend}’$$18:\;\;\;\;\;O_{1}(:,j)\leftarrow K_{1}(idx_{1}(ord_{CM_{2}}),j)$$;\;O_{2}(:,j)\leftarrow K_{2}(idx_{2}(ord_{CM_{3}}),j)$19: **end for**$20:CI_{\mathrm{BP}}(k)\leftarrow subblock(O_{1},k)\;\mathrm{for}\;k=1,\ldots ,4$$;\;CI_{\mathrm{BP}}(k)\leftarrow subblock(O_{2},k-4)\;\mathrm{for}\;k=5,\ldots ,8$$21:idx_{3}\leftarrow \arg_{\mathrm{sort}}(Y_{\mathrm{INT}}[4MN+1:4MN+8])$$22:CI_{\mathrm{BP}}^{\prime }\leftarrow [CI_{\mathrm{BP}}(idx_{3}(1),CI_{\mathrm{BP}}(idx_{3}(2),\ldots ,CI_{\mathrm{BP}}(idx_{3}(8)]$$23:PI_{\mathrm{CON}}\leftarrow \sum_{i=1}^{8}C{I_{\mathrm{BP}}}^{\prime }(i)\times 2^{i-1}$

#### 3.2.2. S-Box-Driven Feedback Diffusion

An S-box without fixed points, reverse fixed points, or short-period cycles can be regarded as a strong S-box, as demonstrated in several existing studies [33,34,35]. In the TCM-IEA, a chaos-based S-box $SB_{\mathrm{TCM}}$ is constructed by utilizing the generated chaotic sequences X, Y, and Z. The $SB_{\mathrm{TCM}}$ is defined as a permutation of integers from 0 to 255. The chaotic sequences are then used to iteratively update $SB_{\mathrm{TCM}}$ through nonlinear transformations and swap operations. The following steps describe the generation process.

**Step 1**: The chaotic sequences X, Y, and Z are generated and reshaped into one-dimensional vectors $X_{c}$, $Y_{c}$, and $Z_{c}$. Meanwhile, $SB_{\mathrm{TCM}}$ is constructed as $SB_{\mathrm{TCM}}=\{0,1,2,\ldots ,255\}$, and a time-varying control parameter is introduced to enhance the dynamic behavior of the system.

**Step 2**: For each element, control parameters are derived from the chaotic sequences, and a nonlinear update mechanism is applied to produce a new state. The updated state is then mapped to an integer index within [1,256] through scaling and modulo operations.

**Step 3**: A swap operation is performed between the current position and the generated index in $SB_{\mathrm{TCM}}$. Meanwhile, the chaotic states are updated through coupling relationships and normalized into the interval [0,1), ensuring continuous evolution of the system.

The above process is iteratively executed for a predefined number of iterations, resulting in a highly nonlinear and complex S-box with an enhanced randomness. The detailed operation is presented in Algorithm 3.
**Algorithm 3**. S-box-driven feedback diffusion**Input**:
X,
Y,
Z,
T,
L**Output**:
$SB_{\mathrm{TCM}}$1: for t=1 **to** T **do**2:      Update control parameter
a and compute mean value of
X
3:    for i=1 **to** 256 **do**$4:\;\;\;\;\;\;\;\;\;\;X(i)=X_{c}(i)$$,\;Y(i)=Y_{c}(i)$$,\;Z(i)=Z_{c}(i)$5:          r=Y(i), A=2×a×r−a, C=2×r, p=Z(i)6:         if p<0.57:               if |A|<1$8:\;\;\;\;\;\;\;\;\;\;\;\;\;\;\;\;\;\;\;\;\;X_{\mathrm{new}}=mean(X)-A\times |C\times mean(X)-X(i)|$9:                **else**10:                     k=(Z(i)modL)+1$11:\;\;\;\;\;\;\;\;\;\;\;\;\;\;\;\;\;\;\;\;\;X_{\mathrm{new}}=X(k)-A\times |C\times X(k)-X(i)|$12:              **end if**13:           **else**$14:\;\;\;\;\;\;\;\;\;\;\;\;\;\;\;\;l_{i}=-1+2Y(i)|$$15:\;\;\;\;\;\;\;\;\;\;\;\;\;\;\;\;X_{\mathrm{new}}=|mean(X)-X(i)|\times \;e^{l_{i}}\times \cos(\pi l_{i})+mean(X)$16:           **end if**$17:\;\;\;\;\;\;\;\;\;\;\;idx_{i}=(floor(|X_{new}|\times 10^{6}+Y(i)\times 10^{6}+Z(i)\times 10^{6})\mathrm{mod}256)+1$$18:\;\;\;\;\;\;\;\;\;\;\mathrm{Swap}\;SB_{\mathrm{TCM}}(i)$$\;\mathrm{and}\;SB_{\mathrm{TCM}}(idx)$19:          Update and normalize X(i), Y(i), Z(i)20:      **end for**21: **end for**$22:\mathrm{Reshape}\;SB_{\mathrm{TCM}}$ into 16×16 S-box$23:\mathrm{return}\;SB_{\mathrm{TCM}}$

To further construct an S-box with a high nonlinearity while eliminating structural weaknesses, an adjustment strategy was employed. Starting from the resulting $SB_{\mathrm{TCM}}$, pairs of elements are randomly exchanged, and only those swaps that improve nonlinearity are retained through iterative updates. Subsequently, fixed points and reverse fixed points are removed via local adjustments. Finally, disjoint cycles are connected into a single long cycle of a 256 length to avoid short periodic structures. The final $SB_{\mathrm{TCM}}$ is tabulated in Table 2. To evaluate its cryptographic performance, several widely used criteria were adopted, including nonlinearity, the bit independence criterion (BIC), the strict avalanche criterion (SAC), and the differential approximation probability (DAP) [36]. The corresponding experimental results are tabulated in Table 3, demonstrating that the S-box achieved a competitive performance.

To further enhance security, a further diffusion operation was introduced to propagate the influence of each pixel over the entire ciphertext.

**Step 1**: The plaintext image is reshaped into a one-dimensional sequence $PI_{\mathrm{vec}}$, and three chaotic sequences X, Y, and Z are quantized and truncated to match the length of $PI_{\mathrm{vec}}$.

**Step 2**: Each element of $PI_{\mathrm{vec}}$ is substituted using the constructed $SB_{\mathrm{TCM}}$ to obtain an intermediate sequence:

(10) $$CI_{3}(i)=SB_{\mathrm{TCM}}(PI_{\mathrm{vec}}(i)).$$

**Step 3**: An initial diffusion value is generated by combining the substituted value and chaotic sequences:

(11) $$CI(1)=CI_{3}(1)⊕X(1)⊕Y(1)⊕Z(1).$$

**Step 4**: A cascading diffusion process is applied, where each ciphertext element depends on the previous one and the chaotic sequences:

(12) $$CI(i)=CI_{3}(i)⊕CI(i-1)⊕X(i)⊕Y(i)⊕Z(i),$$

where i≥2.

### 3.3. Decryption Process

The decryption process, termed the 3D-TCM-based image decryption algorithm (TCM-IDA), is designed as the exact inverse of the proposed TCM-IEA. The original plaintext image can be accurately recovered from the ciphertext using the same secret keys and chaotic sequences.

**Step 1**: The CI is reshaped into a one-dimensional sequence, and the chaotic sequences X, Y, and Z are regenerated and quantized in the same manner as in the TCM-IEA.

**Step 2**: The cascading diffusion process is reversed. The intermediate sequence is obtained as:

(13) $$CI_{3}=CI(i)⊕CI(i-1)⊕X(i)⊕Y(i)⊕Z(i),$$

and for the first element,

(14) $$CI_{3}(1)=CI(1)⊕X(1)⊕Y(1)⊕Z(1).$$

**Step 3**: The initial diffusion is reversed by recovering, which directly corresponds to the substituted sequence before cascading diffusion.

**Step 4**: The inverse S-box substitution is performed using the inverse mapping of $SB_{\mathrm{TCM}}$, which is a bijection over [0,255], to obtain:

(15) $$PI_{\mathrm{vec}}(i)=SB_{\mathrm{TCM}}(CI_{3}(i)).$$

**Step 5**: The sequence $PI_{\mathrm{vec}}$ is reshaped into a two-dimensional image, which corresponds to the permuted image obtained after the bit-level conditional permutation in the encryption stage.

**Step 6**: The inverse bit-level conditional permutation is executed. All operations in Algorithm 2 are reversed in inverse order using the same chaotic index sequences $idx_{1}$, $idx_{2}$, and $idx_{3}$. Specifically, the inverse circular shift is applied with opposite directions, and the inverse sorting operations restore the original positions of rows, columns, and sub-blocks. Finally, the bit-planes are recommended to reconstruct the original plaintext image PI.

Since each component of the encryption process, including XOR-based diffusion, bijective S-box substitution, and index-driven permutation, is strictly invertible, the proposed scheme ensures exact and lossless recovery of the plaintext when the correct key is used.

## 4. Experimental Results and Secure Analysis

**Figure 8:**
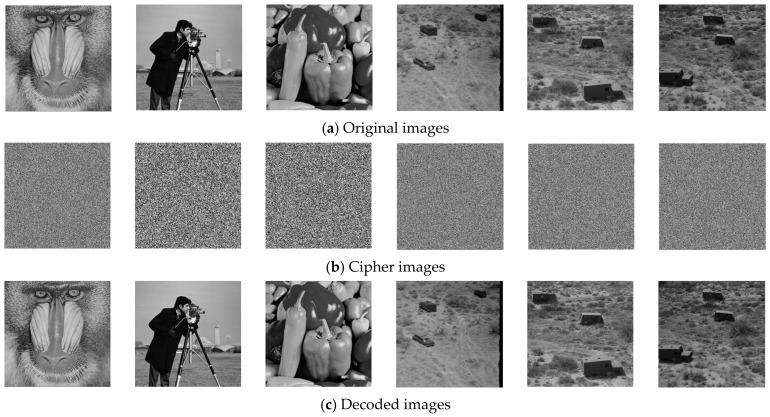
Experimental results of the images.

In this section, a variety of standard grayscale images, primarily selected from the SIPI dataset, are employed to evaluate the performance of the TCM-IEA and TCM-IDA (Figure 8). Comprehensive experimental results are presented, including the histogram distribution, a correlation analysis and the information entropy. In addition, several typical attack scenarios are simulated on the ciphertext images to assess the robustness of the IEA. The results demonstrate that the encrypted images can be correctly recovered, confirming the validity of the decryption process.

### 4.1. Key Space

The secret key of the TCM-IEA consists of three independent components. (1) The initial states and control parameters of the 3D-TCM are represented in double-precision format, with an effective precision of approximately $10^{-15}$. Each parameter contributes about $2^{52}$ possible values. Considering the parameters (a, b, $x_{0}$, $y_{0}$, $z_{0}$), the corresponding key space is approximately ${\left(2^{52}\right)}^{5}=2^{260}$. (2) A hash-based key $HV_{\mathrm{PI}}$ is generated from the plaintext image using the SHA-512 algorithm, providing a key space of $2^{512}$, which introduces strong plaintext dependence. (3) An independent 364-bit random binary sequence is employed, contributing an additional key space of $2^{364}$ and further enhancing the randomness. Since these components are independent, the total key space of the 3D-TCM is sufficiently large to withstand brute-force attacks.

### 4.2. Key Sensitivity

**Figure 9:**
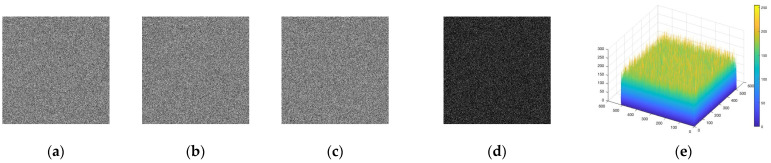
Key sensitivity: the cipher image using (**a**) the original keys, (**b**) $a+10^{-15}$, and (**c**) $HV_{\mathrm{PI}}(2)+1$, (**d**) (**b**–**c**), (**e**) the histogram of (**d**).

**Figure 10:**
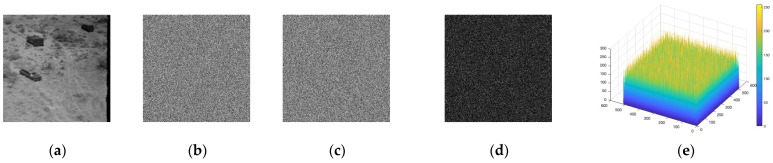
Key sensitivity: the decoded image using (**a**) the original keys, (**b**) $a+10^{-15}$, and (**c**) $HV_{\mathrm{PI}}(2)+1$, (**d**) (**b**–**c**), and (**e**) the histogram of (**d**).

The key sensitivity of the IEA was also evaluated by slightly perturbing the encryption keys [40], altering one bit of either the control parameter a or the key component $HV_{\mathrm{PI}}$ and then executing the encryption and decryption operation. As shown in Figure 9, even tiny changes in the key led to completely different ciphertexts, indicating that the encryption output is highly sensitive to initial key variations. From the decryption perspective, the sensitivity was further verified by attempting to recover the plaintext using slightly altered keys. As illustrated in Figure 10, only the exact original key can correctly reconstruct the plaintext, while any deviation results in meaningless noisy images. This demonstrates that the TCM-IDA is strictly dependent on the correct key and confirms that it serves as the exact inverse of the encryption operation, ensuring the practical realizability and high key sensitivity of the TCM-IDA.

### 4.3. Statistical Analyses

#### 4.3.1. Histogram

**Figure 11:**
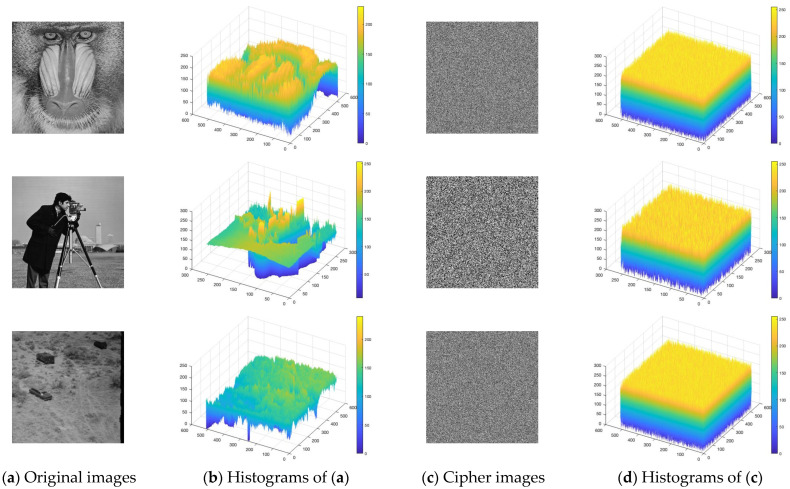
Histograms of the original images and the encrypted images.

A histogram depicts the distribution of pixel intensities in an image and is commonly used to measure its statistical randomness and ability to withstand statistical attacks [41]. As depicted in Figure 11, the original images exhibited non-uniform histograms with prominent peaks, indicating redundant patterns. Their encrypted counterparts instead exhibited a uniform distribution across intensity levels. This demonstrates that the TCM-IEA diffuses the statistical information of the plaintext image, thereby enhancing the resistance to histogram-based statistical attacks.

#### 4.3.2. Correlation

**Figure 12:**
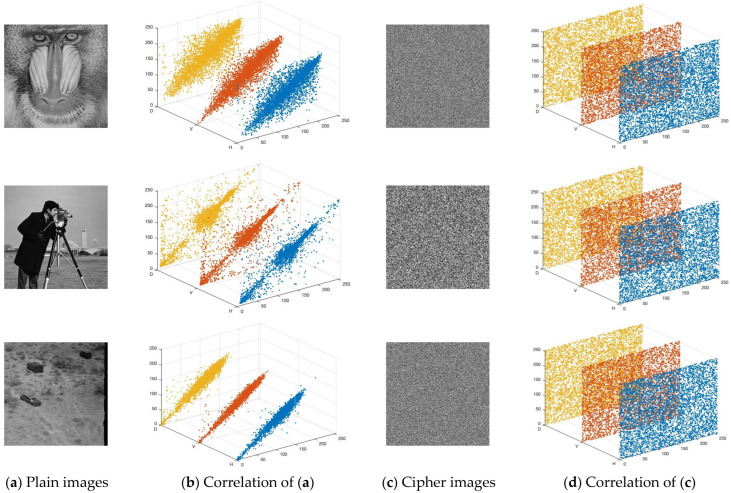
Three-directional correlation of the plain images and their corresponding cipher images.

This section evaluates the capability of the IEA by analyzing the correlation among neighboring pixels in three different directions: horizontally (H), vertically (V) and diagonally (D). The correlation coefficient is calculated by [42]:

(16) $$r_{x,y}=\frac{\sum_{i=1}^{t}\left(x_{i}-E(x))(y_{i}-E(y)\right)}{\sqrt{\sum_{i=1}^{t}{\left(x_{i}-E(x)\right)}^{2}}\sqrt{\sum_{i=1}^{t}{\left(y_{i}-E(y)\right)}^{2}}},$$

where t is the number of pairs of adjacent pixels in the image. E(x) and E(y) are their respective average values. As shown in Table 4 and Figure 12, the plaintext exhibited strong pixel correlation, which the blue, red, and yellow colors represent the three directions. As further observed from Table 4, the TCM-IEA generally achieved correlation coefficients closer to zero than those of Ref. [43], indicating a stronger decorrelation capability across the horizontal, vertical, and diagonal directions. The TCM-IEA can effectively break the pixel correlation.

#### 4.3.3. Information Entropy

Information entropy is a measure of uncertainty for a data source [44]. In IEAs, it determines the randomness in the ciphertext of the image, and thus indicates how secure it is against statistical attacks. The largest entropy attainable for an 8-bit grayscale image that has 256 intensity values is 8, implying a uniform distribution of these 256 values, which gives the greatest level of randomness. It is calculated as follows [45]:

(17) $$H(x)=-\sum_{i=1}^{n}P(x_{i})\log_{2}(P(x_{i})),$$

where n is the total number of pixels. $P(x_{i})$ is the probability of occurrence of gray level $x_{i}$.

Table 5 tabulates the entropy values of the encrypted images, which were consistently close to the ideal value of 8. As can be observed from Table 5, the TCM-IEA achieved entropy values that were comparable to, and in some cases, slightly closer to the ideal value than, those of Refs. [32,43], indicating a more uniform distribution of pixel intensities in the ciphertext. Compared with existing IEAs, the TCM-IEA achieved a competitive performance, confirming its effectiveness against entropy-based attacks.

### 4.4. Differential Attack

Differential attacks assess a cipher’s sensitivity to variations in the plaintext or key inputs, determining if an IEA has a stable avalanche effect. A good IEA produces different ciphertexts no matter how a single pixel is changed in a single image input. The two main metrics that are employed to measure the avalanche effect, named the number of pixel change rate (NPCR) and the unified average changing intensity (UACI) [46], are calculated as follows:

(18) $$NPCR=\frac{\sum_{i=1}^{M}\sum_{j=1}^{N}D(i,j)=\left\{\begin{array}{cc} 1 & \mathrm{if}\;c_{1}(i,j)\neq c_{2}(i,j)\\ 0 & \mathrm{if}\;c_{1}(i,j)=c_{2}(i,j) \end{array}\right.}{M\times N}\times 100\%,$$

(19) $$UACI=\frac{1}{M\times N}\left(\sum_{i=1}^{M}\sum_{j=1}^{N}\frac{\left|c_{1}(i,j)-c_{2}(i,j)\right|}{255}\right)\times 100\%,$$

where $c_{1}(i,j)$ and $c_{2}(i,j)$ are the grayscale intensities at position (i,j). The NPCR measures the proportion of differing pixels between two images encrypted from nearly identical plaintexts, while the UACI quantifies the average intensity difference in those changed pixels. Under uniform randomness, the NPCR and UACI values for grayscale images are approximately 99.6094% and 33.4635%, respectively.

As shown in Table 6, the computed NPCR and UACI scores are close to the ideal values. As further observed from Table 6, the proposed method achieved NPCR and UACI values that were comparable to, and in some cases, slightly better than, those of Refs. [32,43], indicating a strong capability to detect and amplify small changes in the plaintext. This is mainly attributed to the strong diffusion capability of the TCM-IEA, which enables small variations in the plaintext to rapidly propagate across the entire ciphertext image, resulting in near-uniform statistical distributions. The results demonstrate that the TCM-IEA achieves a strong resistance against differential attacks. In addition, the standard deviations of NPCR and UACI across different test images were small, which further confirms the stability and statistical reliability.

### 4.5. Noise and Data Loss Attacks

**Figure 13:**
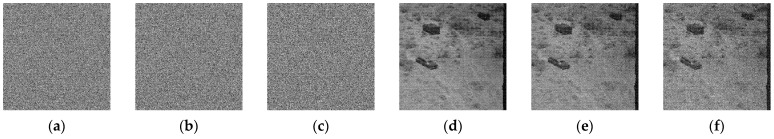
Noise resistance: (**a**) 0.01-, (**b**) 0.05-, and (**c**) 0.1-intensity salt and pepper of the encrypted image 5.1.09; (**d**–**f**) are the decoded images of (**a**–**c**).

**Figure 14:**
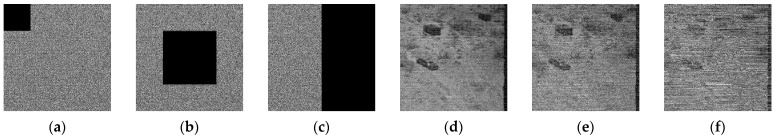
Data loss: (**a**) 1/16, (**b**) 1/4, and (**c**) 1/2 of the encrypted image 5.1.09; (**d**–**f**) are the decoded images of (**a**–**c**).

For an IEA, their cipher images should remain robust against the distortion from transmission or storage [47]. This section tests the TCM-IEA against two main indicators: salt-and-pepper noise at different densities and cropped attacks at different ratios. The restored images presented in Figure 13 and Figure 14 still preserve the intact structural information and visual details, even though they have undergone significant distortions due to the introduction of large amounts of random noises and missing small blocks.

### 4.6. NIST Test for Encrypted Images

To further validate the randomness of the TCM-IEA, the NIST SP800-22 test suite was also applied to the ciphertext sequences. Specifically, the encrypted images were first converted into one-dimensional binary sequences and then evaluated using the same 15 statistical tests as described in Section 2.6. As shown in Table 7, the *p*-values obtained from all the tests were greater than the significance level of 0.01, indicating that the null hypothesis of randomness cannot be rejected. In addition, for tests such as the non-overlapping template, random excursions, and random excursions variant, the pass ratios satisfied the required criteria, demonstrating stable statistical behavior. Compared with the results of chaotic sequences, the ciphertext sequences also exhibited consistent randomness across all the tested dimensions. This indicates that the TCM-IEA not only preserves the randomness of the underlying 3D-TCM, but also effectively propagates it through the permutation–diffusion process. Therefore, the NIST test results on the encrypted images further confirm that the TCM-IEA produces ciphertexts with a high statistical randomness, providing strong support for its security and resistance.

### 4.7. Computational Complexity

The computational complexity of the TCM-IEA mainly consists of three stages: key generation, bit-level permutation, and S-box-based feedback diffusion. In the first stage, the SHA-512 hash computation requires O(MN) operations, where M and N denote the image dimensions. The subsequent parameter update and chaotic sequence generation involve linear iterations proportional to the image size, resulting in an overall complexity of O(MN). In the bit-level permutation stage, the image is decomposed into eight bit-planes and processed through index-based rearrangement, circular shifts, and sorting operations. Since these operations involve linear traversal and local sorting within fixed-size structures, the overall complexity remains O(MN). In the diffusion stage, each pixel undergoes S-box substitution and feedback-based XOR operations with chaotic sequences. As these operations are performed sequentially over all pixels, the computational complexity is also O(MN). Therefore, the overall computational complexity of the TCM-IEA is O(MN), which is consistent with most existing chaos-based IEAs.

## 5. Conclusions

The TCM-IEA combines a three-dimensional CM with a plaintext-related key generation mechanism to achieve a strong security performance. By integrating the SHA-512 hash function with parameter reconstruction, the TCM-IEA enhances the key sensitivity and establishes a strong dependency between the plaintext and the generated keys. The introduction of a dynamically generated S-box further improves nonlinearity, while the bit-level permutation and feedback diffusion mechanisms effectively strengthen the confusion and diffusion properties. The experimental results demonstrate that the TCM-IEA achieved excellent statistical characteristics, including a high information entropy, a low pixel correlation, and a strong resistance against differential attacks. In addition, the TCM-IEA shows robustness under various attack scenarios such as noise and data loss, confirming its reliability in practical applications. The overall computational complexity is O(MN), indicating that the TCM-IEA is efficient and suitable for image encryption tasks.

## Figures and Tables

**Table 1:** NIST test results.

No.	Item	Sequence					
		*x*		*y*		*z*	
1	Approximate entropy	0.7545	*p* *	0.3071	*p*	0.2980	*p*
2	Block frequency	0.6899	*p*	0.5948	*p*	0.2804	*p*
3	Cumulative sums—forward	0.4891	*p*	0.1024	*p*	0.7329	*p*
	Cumulative sums—reverse	0.5095	*p*	0.0737	*p*	0.8156	*p*
4	FFT	0.8942	*p*	0.3636	*p*	0.2672	*p*
5	Frequency	0.3384	*p*	0.0981	*p*	0.9284	*p*
6	Linear complexity	0.2891	*p*	0.1458	*p*	0.0296	*p*
7	Longest run	0.6785	*p*	0.0537	*p*	0.7850	*p*
8	Non-overlapping template **	148/148	– ***	148/148	–	143/148	–
9	Overlapping template	0.1510	*p*	0.7605	*p*	0.0330	*p*
10	Random excursions **	8/8	–	8/8	–	7/8	–
11	Random excursion variant **	18/18	–	18/18	–	18/18	–
12	Rank	0.3101	*p*	0.9316	*p*	0.8059	*p*
13	Runs	0.3231	*p*	0.6583	*p*	0.0370	*p*
14	Serial test *p*-value 1	0.7978	*p*	0.2079	*p*	0.7437	*p*
	Serial test *p*-value 2	0.2612	*p*	0.2424	*p*	0.2990	*p*
15	Universal	0.4090	*p*	0.8926	*p*	0.9097	*p*

* “*p*” denotes that the sequence passes the test at the significance level of 0.01. ** For the non-overlapping template, random excursions, and random excursion variant tests, the results are tabulated as the number of passed subtests over the total number of tests. *** “–” indicates that no separate pass or fail indicator is tabulated for this test. The result is expressed as a pass ratio.

**Table 2:** Constructed results of the S-box.

	0	1	2	3	4	5	6	7	8	9	A	B	C	D	E	F
0	B8	8F	4E	C1	44	CC	38	DA	BA	E7	1B	F7	08	77	95	49
1	98	70	9C	26	A0	A7	A5	61	AB	5B	B1	B7	D6	4B	C3	41
2	CE	36	52	29	06	DC	F6	56	19	79	F0	76	0E	71	9A	93
3	2E	68	11	2A	A9	85	AF	96	12	4F	BE	46	C8	3C	D2	D7
4	DE	24	EB	16	FA	05	5F	AD	58	B4	9B	BD	47	89	51	D3
5	30	DF	23	EC	15	FB	6E	69	62	F2	AA	5A	B3	3D	BC	45
6	C6	3B	D1	2F	DD	25	21	17	F9	07	7B	90	78	9D	74	50
7	6C	A1	64	67	D9	B0	72	B9	20	C4	3F	CF	83	D8	28	E4
8	1E	F1	10	0F	01	88	F5	09	81	80	5C	0A	7D	8E	7A	1A
9	92	99	E9	9F	1D	A6	BF	E0	55	6D	4A	C0	42	CA	39	D4
A	7C	73	27	E3	1F	EF	B6	FC	FF	5E	AC	53	BB	48	C5	3E
B	CD	33	91	34	E2	0D	ED	13	F4	0B	31	6F	97	66	A2	57
C	B5	4C	40	CB	35	2B	2C	E1	63	E6	54	A4	DB	FE	18	A3
D	FD	02	14	F8	04	2D	EE	65	8B	03	82	94	32	B2	75	59
E	60	C9	43	D5	37	6B	9E	E5	22	F3	0C	7F	C2	6A	D0	3A
F	1C	AE	E8	5D	8C	84	8D	EA	A8	4D	8A	86	0	87	C7	7E

**Table 3:** Comparative performance of the TCM-IEA and existing IEAs.

S-Box	Nonlinearity			BIC	SAC	DAP
	Min	Max	Mean			
Ours	104	108	106.25	102.79	0.5078	0.0391
AES	112	112	112	112	0.5048	0.0156
SM4	112	112	112	112	0.4997	0.0156
Ref. [37]	100	108	105	103	0.5002	0.0469
Ref. [38]	100	108	104	103.5	0.516	0.039
Ref. [39]	98	106	103.5	103.93	0.4958	0.313

**Table 4:** Correlation coefficient results.

Test Image	Ours						Ref. [43]		
	Plain Image			Cipher Image			Cipher Image		
	H	V	D	H	V	D	H	V	D
Baboon	0.8665	0.7586	0.7261	−0.0012	−0.0002	0.0009	0.0018	−8.3384 × 10^−4^	−0.0012
Cameraman	0.9335	0.9592	0.9087	0.0014	0.0027	0.0043	0.0023	−0.0014	−0.0039
Peppers	0.9659	0.9636	0.9325	−0.0013	0.0006	−0.0004	0.0026	0.0046	−0.0046

**Table 5:** Entropy values.

Image	IES		
	Ref. [32]	Ref. [43]	Ours
7.1.01	7.9993	7.9994	7.9993
7.1.02	7.9991	7.9993	7.9992
7.1.03	7.9993	7.9993	7.9994
7.1.04	7.9994	7.9993	7.9993
7.1.05	7.9992	7.9994	7.9992
7.1.06	7.9993	7.9993	7.9993

**Table 6:** NPCR(%) and UACI(%) values.

Image	NPCR			UACI		
	Ref. [32]	Ref. [43]	Ours	Ref. [32]	Ref. [43]	Ours
7.1.01	99.6040	99.6262	99.6193	33.4694	33.4223	33.4834
7.1.02	99.6212	99.6147	99.6162	33.4410	33.5315	33.4065
7.1.03	99.6151	99.6193	99.6006	33.4801	33.4990	33.4192
7.1.04	99.6014	99.6025	99.6044	33.4845	33.4838	33.4994
7.1.05	99.6109	99.5956	99.6052	33.4939	33.4713	33.4661
7.1.06	99.6124	99.5983	99.6189	33.4338	33.4577	33.5128
Mean	99.6140	99.6147	99.6108	33.4663	33.4615	33.4646

**Table 7:** NIST test results of the cipher images.

No.	Item	Cipher Image					
		7.1.02		7.1.03		7.1.04	
1	Approximate entropy	0.6495	*p*	0.5052	*p*	0.4894	*p* *
2	Block frequency	0.9344	*p*	0.6135	*p*	0.4107	*p*
3	Cumulative sums—forward	0.8381	*p*	0.2279	*p*	0.665	*p*
	Cumulative sums—reverse	0.5882	*p*	0.1544	*p*	0.4046	*p*
4	FFT	0.3204	*p*	0.6907	*p*	0.4693	*p*
5	Frequency	0.5243	*p*	0.1362	*p*	0.7539	*p*
6	Linear complexity	0.7722	*p*	0.9718	*p*	0.0963	*p*
7	Longest run	0.2945	*p*	0.7785	*p*	0.5604	*p*
8	Non-overlapping template **	145/148	– ***	145/148	–	147/148	–
9	Overlapping template	0.6729	*p*	0.7003	*p*	0.8515	*p*
10	Random excursions **	8/8	–	8/8	–	8/8	–
11	Random excursion variant **	17/18	–	18/18	–	18/18	–
12	Rank	0.2574	*p*	0.3435	*p*	0.5741	*p*
13	Runs	0.6038	*p*	0.6626	*p*	0.9636	*p*
14	Serial test *p*-value 1	0.5463	*p*	0.086	*p*	0.2642	*p*
	Serial test *p*-value 2	0.4193	*p*	0.4075	*p*	0.041	*p*
15	Universal	0.293	*p*	0.7286	*p*	0.5081	*p*

* “*p*” denotes that the image passes the test at the significance level of 0.01. ** For the non-overlapping template, random excursions, and random excursion variant tests, the results are tabulated as the number of passed subtests over the total number of tests. *** “–” indicates that no separate pass or fail indicator is tabulated for this test. The result is expressed as a pass ratio.

## Data Availability

The original contributions presented in this study are included in the article. Further inquiries can be directed to the corresponding author.
